# In Silico Structural Analysis Exploring Conformational Folding of Protein Variants in Alzheimer’s Disease

**DOI:** 10.3390/ijms241713543

**Published:** 2023-08-31

**Authors:** Evangelos Efraimidis, Marios G. Krokidis, Themis P. Exarchos, Tamas Lazar, Panagiotis Vlamos

**Affiliations:** 1 Bioinformatics and Neuroinformatics MSc Program, Hellenic Open University, 26335 Patras, Greece; std513877@ac.eap.gr; 2 Bioinformatics and Human Electrophysiology Laboratory, Department of Informatics, Ionian University, 49100 Corfu, Greece; mkrokidis@ionio.gr (M.G.K.); exarchos@ionio.gr (T.P.E.); 3 VIB–VUB Center for Structural Biology, Vlaams Instituut voor Biotechnologie (VIB), B1050 Brussels, Belgium; tamas.lazar@vub.be; 4 Structural Biology Brussels, Department of Bioengineering, Vrije Universiteit Brussel, B1050 Brussels, Belgium

**Keywords:** protein folding, functional analysis, Alzheimer’s disease, presenilin 1, fold recognition

## Abstract

Accurate protein structure prediction using computational methods remains a challenge in molecular biology. Recent advances in AI-powered algorithms provide a transformative effect in solving this problem. Even though AlphaFold’s performance has improved since its release, there are still limitations that apply to its efficacy. In this study, a selection of proteins related to the pathology of Alzheimer’s disease was modeled, with Presenilin-1 (PSN1) and its mutated variants in the foreground. Their structural predictions were evaluated using the ColabFold implementation of AlphaFold, which utilizes MMseqs2 for the creation of multiple sequence alignments (MSAs). A higher number of recycles than the one used in the AlphaFold DB was selected, and no templates were used. In addition, prediction by RoseTTAFold was also applied to address how structures from the two deep learning frameworks match reality. The resulting conformations were compared with the corresponding experimental structures, providing potential insights into the predictive ability of this approach in this particular group of proteins. Furthermore, a comprehensive examination was performed on features such as predicted regions of disorder and the potential effect of mutations on PSN1. Our findings consist of highly accurate superpositions with little or no deviation from experimentally determined domain-level models.

## 1. Introduction

For globular proteins to achieve biological activity, the appropriate, thermodynamically stable three-dimensional state needs to be reached. Lately, the prediction of the three-dimensional structure of proteins directly from their amino acid sequence is dominating biomedical research [1,2,3]. Over 50 years have passed since Anfinsen’s refolding experiments established that there are intrinsic properties in a protein’s amino acid sequence, which ultimately determine its three-dimensional structure [4]. To this day, there is an ongoing effort to clarify sequence–structure relationships in proteins with varying levels of success. However, accurately predicting the structure of the protein when no known templates are available has always been a very difficult exercise [5]. The application of deep learning algorithms for this purpose has become a promising prospect over the past decade, resulting in increasingly better predictions [6]. Finally, with the recent advancements presented in CASP14 by DeepMind and their model AlphaFold2, researchers now have tools at their disposal to achieve highly accurate structural predictions even when there are very little to no known homologous structures [7]. The ability to accurately predict protein conformation is of enormous benefit to the scientific community, greatly accelerating efforts to understand the building blocks of cells and enabling faster and more advanced drug discovery.

For the past two years, AlphaFold2 has been in the foreground of discussions and benchmarking efforts for protein structure prediction [8,9]. Recently, the AlphaFold database holds over 214 million structures, whereas the total number of experimental protein data bank (PDB) entries remains around 200 thousand [10]. Nevertheless, limitations remain, and given the use of these approaches, validation of their predictive capabilities is arguably more important than ever. AlphaFold was designed to predict one or a few conformations for proteins, therefore providing locally lower quality and less realistically looking results in molecules that have a dynamic conformation, such as intrinsically disordered regions or long unstructured loops. Furthermore, it is currently only compatible with proteins of unmodified polypeptide chains, hence it does not give clear answers for multi-chain protein complexes, including multiple protein-DNA and protein-small molecule interactions, as the predicted model may reflect the apo or the holo form for a protein that binds partners with conformational change, which ambiguity can only be resolved for cases with experimental evidence. Another notable feature of AlphaFold is that its per-residue confidence metric, the predicted Local Distance Difference Test (pLDDT) score, is often a good indication of the intrinsic disorder [11].

ColabFold (versions 1.3.0 and 1.5.2) is an open-source software based on the AlphaFold model that can be accessed and used within the Google Collaboratory environment as a Jupyter Notebook [12]. Similar to the AlphaFold version developed by DeepMind, ColabFold provides a convenient way for researchers to carry out protein structure prediction tasks through a personal computer, without the need for specialized hardware. One key feature of ColabFold is its use of MMseqs2 (version edb822), a software program for rapid many-against-many sequence searching [13] instead of HMMer [14] during the pre-processing step. This substitution significantly reduces the time required for homology searching and improves the speed of MSA generation, with no adverse impact on the accuracy of structure prediction [9]. RoseTTAFold is another structure prediction method that has received major recognition due to its high accuracy. Through the employment of a “three-track” deep learning network, the algorithm can predict high-quality tertiary structures and is also able to generate accurate models for protein complexes [15]. The “three-track” network achieves remarkable performance by processing and combining information relative to the amino acid sequence, the residue-residue distances, and the atomic coordinates. RoseTTAFold is freely accessible and can be run through the Robetta web server [15].

Proteins evolve highly specific sequences to adopt a native structure that is optimized to efficiently perform their functions. Protein misfolding or structural destabilization, caused by single point mutations or external factors, and the related accumulation of protein aggregates may cause various pathological processes, such as neurodegenerative disorders [16]. Protein misfolding and subsequent amyloid aggregation is a risk marker for Alzheimer’s disease (AD), the most common cause of neurodegenerative dementia in the elderly, which is characterized by progressive cognitive impairment [17]. Approximately 1–2% of the disorder is inherited in an autosomal dominant manner as a consequence of mutations in the Amyloid Precursor Protein (APP) genes, presenilin 1 (PSN1) or 2 (PSN2) [18]. Furthermore, the Apolipoprotein E (APoE) genotype is included in the major risk factors for AD progression [19]. The neuropathological hallmarks of the disease are neuritic plaques and neurofibrillary tangles (NFTs) [20,21]. More specifically, extracellular deposition of β-amyloid (Aβ) in the form of diffuse plaques and the presence of intracellular NFTs and neuropil helical filaments within dystrophic neurites consisting of aggregated hyperphosphorylated tau protein [22]. These lead finally to the loss of synapses and neurons in vulnerable areas that strongly characterize the symptoms of AD, with the predominant one being dementia [23]. APP is a membrane protein expressed in many tissues, especially at the synapses of neurons. It is synthesized in the endoplasmic reticulum and then transported to the Golgi complex, where its maturation is completed and finally transferred to the plasma membrane. The protein is cleaved by the presence of β-secretases and γ-secretases to produce the Aβ, a 37 to 49 amino acid peptide, and its amyloid fibrin form is the main component of the amyloid plaques found in the brains of AD patients [24]. In neuronal tissue, the non-canonical isoform APP695 (UniProt accession: P05067-4) is predominant [25].

The present work focused on PSN1 conformation through the analysis of a set of mutated variants that are ranked pathogenic for AD. Herein, ColabFold was used as an implementation of the AlphaFold framework that utilizes the MMseqs2 algorithm to quickly compile multiple sequence alignments (MSAs). RoseTTAFold was also applied to address to what extent the models from the two deep learning methods accord with each other. To be able to generalize these conclusions, a few more proteins implicated in AD were also included in the analysis to verify and cross-compare their conformations between the resulting computational models and experimental PDB structures. After superposition, the TM-score and RMSD metrics of C_a_ atoms were estimated and illustrated using PyMOL and the TM-align algorithm [26]. In addition, a comprehensive look into the superposed structures was performed to observe any large deviations between pairs of residues. Furthermore, various computational methods were employed to address the concepts of intrinsic protein disorder, recognition of binding residues, and the effect of missense mutations in protein stability and structure prediction.

## 2. Results

For each of the four proteins of interest, five structural models were generated through the ColabFold implementation of the AlphaFold algorithm by introducing the specified parameters, including a higher number of recycles than the default. RoseTTAFold models were also generated using the default parameters built into the framework that runs on the web server. The direct output consists of five structural models, with each model accompanied by a plot of the estimated error for each residue measured in Å (Figures S11–S14). These models were used to evaluate the performance of the two deep learning-based structure prediction tools. This evaluation focused on both global and local deviations from experimentally resolved structures of the AD-related proteins (Section 2.1 and Section 2.2). Furthermore, the modeling effort enabled us to assess the missing segments of these proteins that far not resolved by the experimental structures. For the catalytically important exons 8–9 of PSN1, we also evaluated the likely effect of pathogenic missense mutations (Section 2.3). Through disorder predictions, we demonstrate that the missing segments of PSN1 and the other AD-related proteins are too mobile to capture by X-ray crystallography or cryo-electron microscopy—at least in the absence of stabilizing partners fixing these intrinsically disordered regions (IDRs) (Section 2.4). Lastly, we show known and propose new putative binding motifs within these IDRs for future study.

### 2.1. Comparison Metrics for PSN1, APOE, APP695 and TREM2

The proteins under investigation, namely PSN1, APOE, APP695, and TREM2, are known to fulfill important roles in the pathogenesis of AD. The following subsections focus on the presentation and analysis of comparison metrics derived from structural superpositions of the predicted models and their corresponding reference structures sourced from the PDB. Superpositions regarding PSN1 are thoroughly analyzed and discussed in a later chapter. The resulting comparison metrics are encapsulated in Table 1, and snapshots of the superposed structures are provided in Figures S1–S4.

#### 2.1.1. Apolipoprotein E (APOE)

**Figure 1:**
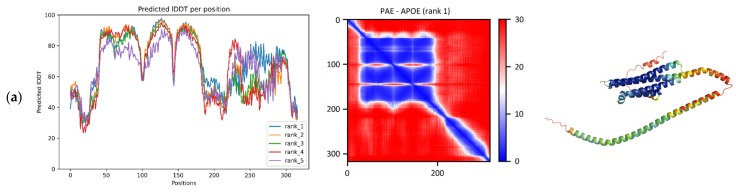

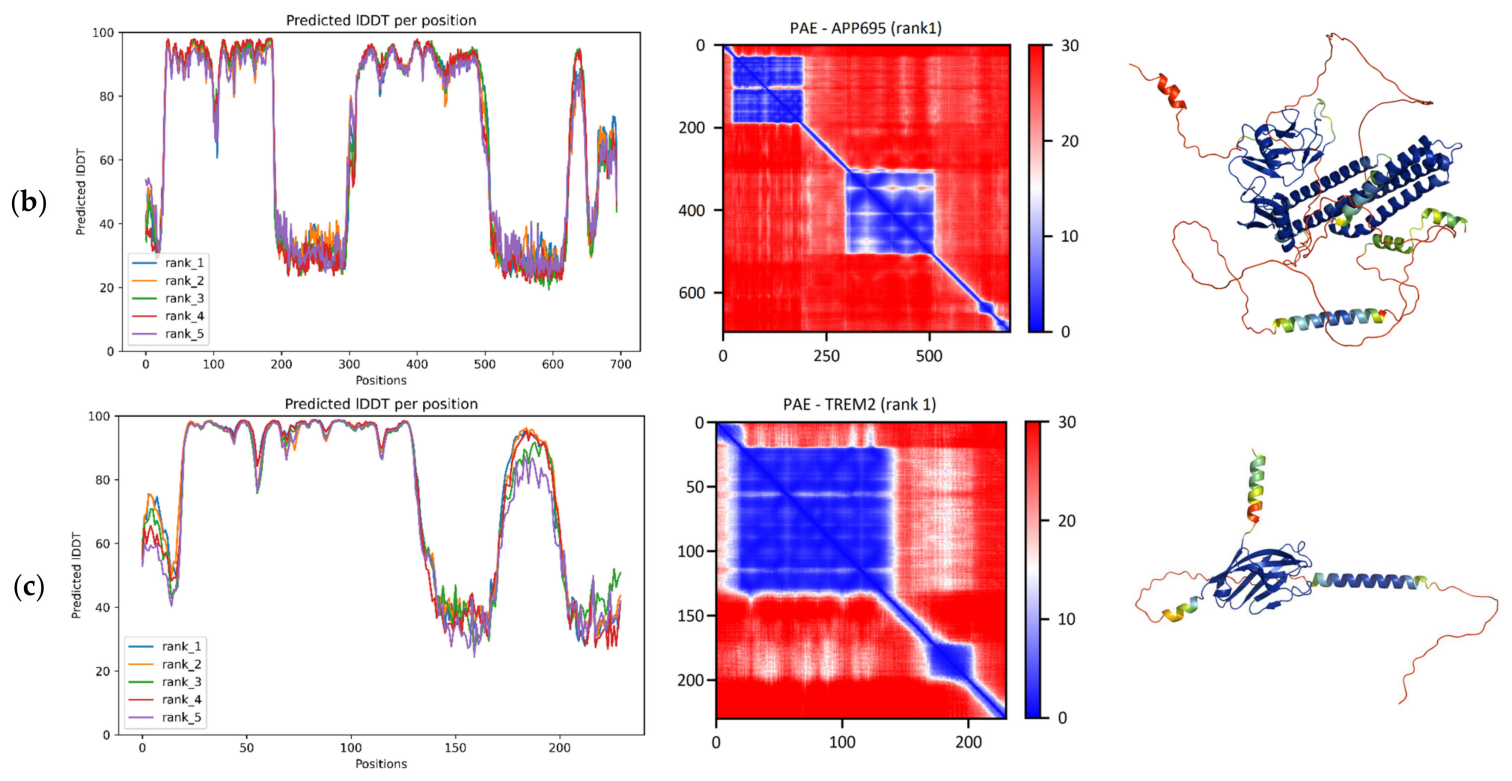
pLDDT and PAE plots produced by ColabFold when executed on the sequences of the four selected proteins (**a**) APOE (P02649), (**b**) APP695 (P05067-4), (**c**) TREM2 (Q9NZC2). The pLDDT plots are on the (**left**) column. PAE matrices in the (**middle**) column, and snapshots of the 3D structures colored by pLDDT are shown on the (**right**) column. In the pLDDT plots, the x-axis corresponds to residues, and the y-axis to the pLDDT value. Different colors correspond to the five different models generated by the algorithm. In the PAE matrices, both axes correspond to residues, and the color represents the value of the PAE metric. Only the PAE matrix and model snapshot of the best (according to pLDDT) out of the five models is presented for each protein.

According to the pLDDT plot, a large region consisting of a series of four smaller consecutive regions was provided with high confidence among the residues 43–178, as Figure 1a shows. The PAE matrix suggests that the relative positions of these smaller regions are also given with high confidence (Figure 1a). In the predicted model, four alpha-helices are highlighted, connected to each other by turns and a small (8 residue) helix. Together, they form a four-helical bundle that belongs in the “Hemerythrin-type up-and-down 4-helical bundle” fold (SCOP ID: 2000080). The reference structure 7FCR has the same four-helical bundle fold. Running the TM-align algorithm on the ColabFold model and the reference structure produces an adequate superposition with a TM-score of 0.96 (range: 0.95–0.97) and a median RMSD of 1.41 Å (range: 0.86–0.44) (Table 1). In like manner, superposing the five RoseTTAFold models and the reference structure resulted in a median TM-score of 0.95 (range: 0.94–0.95) and a median RMSD of 1.11 Å (range: 1.07–1.28) (Table 1).

The C-terminal region, on the other hand, obtained lower pLDDT scores, and these scores deviate a lot more across the top 5 models, indicative of some modeling ambiguity of the region. The PAE matrix of this protein does not indicate the interplay between the N- and the C-terminal regions, although the literature has proposed that the C-terminus may not always be in an extended conformation, but the protein is capable of forming a more compact structure in a small fraction of its conformational ensemble [27].

#### 2.1.2. Isoform APP695 of Amyloid-Beta Precursor Protein (APP695)

For APP, the major isoform APP695 was examined, as it is the predominant isoform in neuronal tissue. Two differences distinguish this isoform’s sequence from the canonical isoform: Glu 289 is replaced by Val, and the 75-residue segment 290–364 is absent. The resulting pLDDT plot indicates the existence of a region predicted with high confidence in the amino acid range of 30–188 (Figure 1b). Another high-confidence region consisting of multiple smaller ones is found roughly along the residues 311–500 (corresponding to the 386–575 region of the canonical sequence). The relative positions of residues within each of the two aforementioned regions are predicted with high confidence according to the PAE matrix, as Figure 1b shows. However, the relative position between the two regions may be unreliable. Lastly, a very small region of high confidence is found at residues 628–648 (703–723 on the canonical sequence). As seen in the resulting model, the domain at 30–188 (known as the E1 domain of APP) consists of two subdomains. The first region (30–120) is the GFLD subdomain, and it adopts a specific “SRCR-like” fold (SCOP ID: 2000834) with a beta(5)-alpha-beta-loop-beta motif. The second region (131–188) is the CuBD subdomain, and it adopts a “Dodecin subunit-like” fold (SCOP ID: 2000724) with a beta-alpha-beta(2) motif. The next high-confidence region (311–500) is predicted as five helices in a “STAT-type 4-helical bundle” fold (SCOP ID: 2000094) that is followed by an additional helix. The small region (628–648) near the C-terminal end of the structure is predicted as a single helix. It belongs to the amyloid beta peptide, which operates as an anchor transmembrane domain for APP. However, it is the same peptide that can aggregate and form amyloid fibrils and plaques that accumulate and lead to neurodegeneration. The provided ColabFold model and the reference structures 4PQD, 2FMA, 1TKN, and 2LLM were superposed, and the TM-scores and RMSDs were calculated. Interestingly, this 21-residue-long helix has not been observed as a very long helix in experimental structures, but the first 12 residues of this helix are usually seen as coil-like (PDBe-Kb: P05067/structures [28].

To evaluate the AI-based structure predictions, the five ColabFold and RoseTTAFold models were superposed against the reference structures 4PWQ and 1TKN, and the TM-score and RMSD metrics were calculated. The resulting metrics suggest overall high accuracy on the domain level. The reference 4PWQ resolves the first two subdomains at aa. 28–189. The ColabFold structures were superposed with a median TM-score equal to 0.95 (range: 0.94–0.95) and a median RMSD of 1.31 Å (range: 1.27–1.52) (Table 1). For this reference structure, the five RoseTTAFold superpositions had a median TM-score of 0.94 (range: 0.93–0.94) and a median RMSD of 1.54 Å (range: 1.38–1.64) (Table 1), which appears to be a slightly worse overall result compared to ColabFold. For the 4-helical bundle region, the PDB entry 1TKN resolves aa. 460–569 was used as a reference. The five ColabFold superpositions had a median TM-score of 0.77 (range: 0.77–0.78) and a median RMSD of 2.58 Å (range: 2.52–2.69) (Table 1). Here, superposing the RoseTTAFold structures with the reference led to a median TM-score of 0.81 (range: 0.76–0.82) and a median RMSD of 2.35 Å (range: 2.23–2.71) (Table 1). The pLDDT scores in the regions falling outside these domains are significantly lower, and thus are indicative of IDRs, especially considering the fact that these regions have never been experimentally resolved despite the serious focus on understanding the structure of these proteins (signified by a total of 183 PDB structures available for APP and its isoforms). These interdomain IDRs and the disordered C-terminus may serve as linear motif display sites or entropic chains (linkers, spacers) between domains. For more details on these regions, see Section 2.4.

#### 2.1.3. Triggering Receptor Expressed on Myeloid Cells 2 (TREM2)

As seen in the pLDDT plot for TREM2’s analysis, there is a large region of very high confidence in the residue range 20–133 and a smaller one in 173–198 (Figure 1c). According to the PAE matrix, the relative position of residues within each of these regions is confidently predicted as well. Looking at the resulting structure reveals that the first region of confidence is given as a domain consisting of 9 beta strands that form a beta-sandwich of the “Immunoglobulin-like beta-sandwich” fold (SCOP ID: 2000051). The small region near the C-terminal end is predicted as a single alpha-helix, as Figure 1c indicates. Comparison of the five ColabFold models and the reference structure 5UD8 (chain B), resolving TREM2 at aa. 19–130 reveals another set of highly accurate superpositions. This comparison regards the beta-sandwich domain located in the 20–133 region. The resulting metrics were extremely consistent among the five models, with the TM-score being 0.94 in all superpositions and the median RMSD being 1.55 Å (range: 1.54–1.56) (Table 1). Upon superposition of the five RoseTTAFold models, the median TM-score was a bit lower at 0.91 (range: 0.90–0.92), and the median RMSD was also slightly worse at 1.83 Å (range: 1.79–1.90) (Table 1). This result in itself suggests a fine match between the structures, but a second comparison with another reference structure (5ELI, chain A) from a distinct PDBe-Kb cluster exhibited better scores for the same domain (residues 19–133). The superposition of the ColabFold model resulted in a median TM-score of 0.98 (range: 0.97–0.98) and median RMSD of 0.6 Å (range: 0.57–0.66) (Table 1). Using the five RoseTTAFold structures led to slightly lower but still highly adequate results, with the median TM-score being 0.95 (range: 0.94–0.95) and the median RMSD 0.98 Å (range: 0.92–1.01) (Table 1). The superposition results indicate that both algorithms are picking up the latter cluster conformation represented by 5ELI, which mainly differs from 5UD8 in its N-terminus and the partially resolved unstructured loop in 5UD8 at aa. 66–81 that is fully resolved in 5ELI.

### 2.2. Presenilin-1 (PSN1)

**Figure 2:**
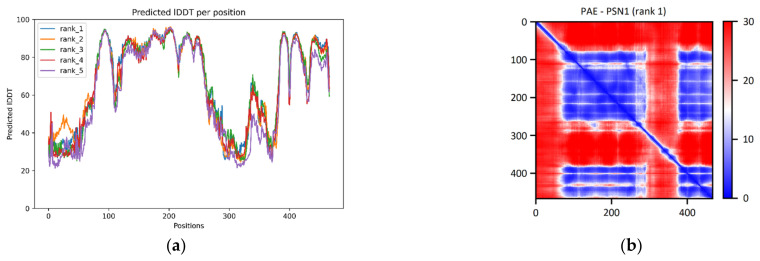

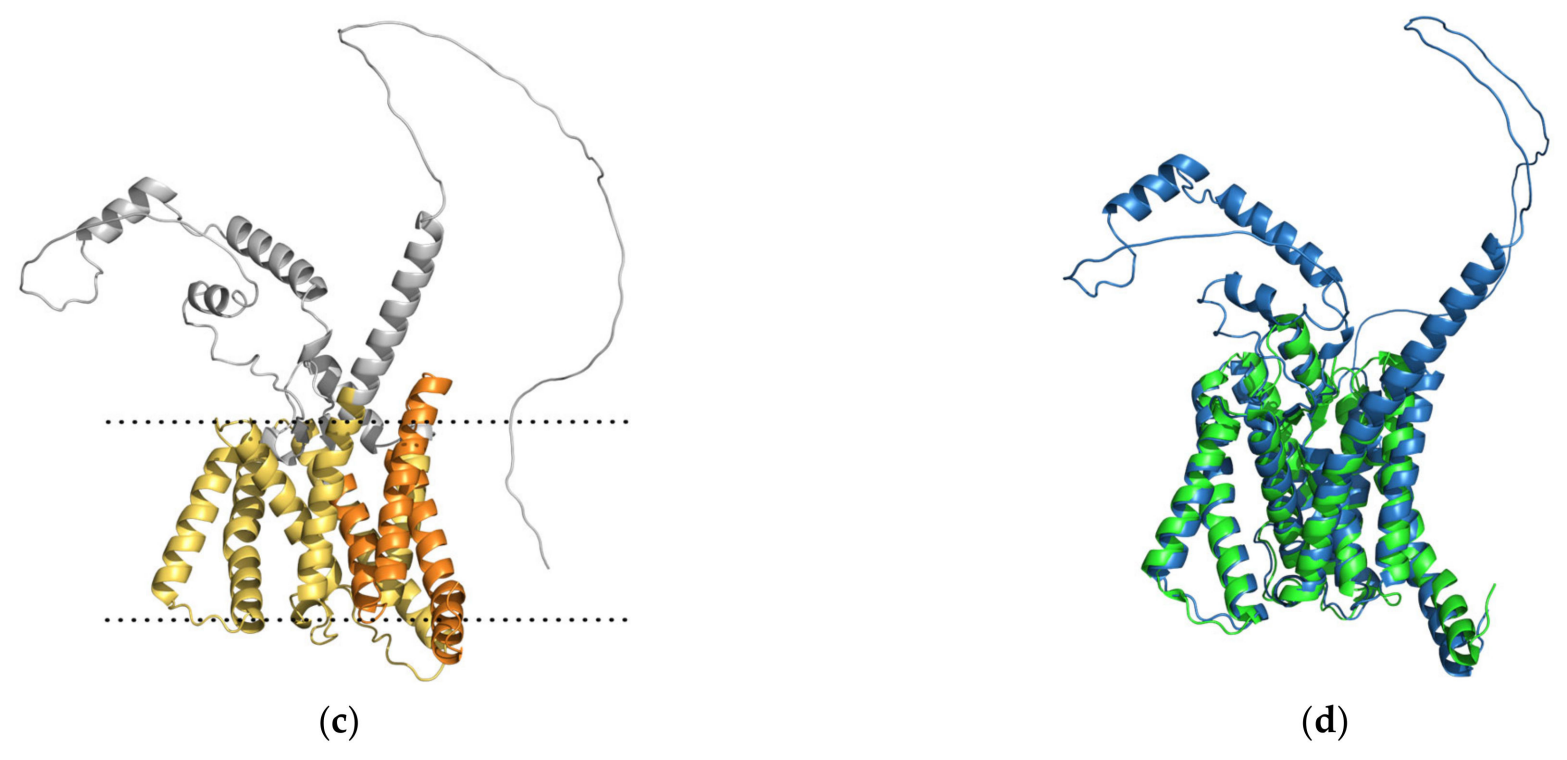
ColabFold analysis for PSN1 (P49768). (**a**) In the pLDDT plot, the x-axis corresponds to residues, and the y-axis to the pLDDT value. Different colors correspond to the five different models created by the algorithm. (**b**) In the PAE matrix, both axes correspond to residues, and the color represents the value of the PAE metric. Only the PAE matrix of the best (according to pLDDT) out of the five models is presented for each protein. (**c**) ColabFold structural model of PSN1. The yellow region corresponds to aa. 80–260, orange region corresponds to aa. 385–465. The two-colored regions make up the transmembrane part of the protein. The dotted-lines represent the lipid bilayer, with the upper part corresponding to the extracellular space. (**d**) Structural superposition of the wild-type ColabFold model (in blue) and the reference PDB structure 7D8X (in green). Structural images were generated using PyMOL.

Examination of the pLDDT plot derived from the PSN1 prediction suggests the existence of two high-confidence regions. The first one is located between residues 80 and 260, and the second is at the C-terminal end of the chain, between residues 385 and 465 (Figure 2a). According to the PAE matrix, these two regions are also given with high confidence in their relative positions. Both of the regions identified in the plots can be seen in the final model as a complex formation of alpha-helices. These helices compose the transmembrane part of the protein (Figure 2c). Comparing the ColabFold model with the corresponding structure of the online AlphaFold database produces a superposition with a TM-score of 0.74 and an RMSD of 2.57 Å. This deviation is caused purely by the misalignment of the low pLDDT regions. However, all the high-confidence regions are precisely aligned. Upon comparison of the ColabFold model to the corresponding chain of the reference structure 7D8X (chain B, resolving PSN1 at aa. 76–291 and 377–467) via the TM-align algorithm, the superpositions produced present a TM-score of 0.91 (range: 0.88–0.94) and a median RMSD of 2.19 Å (range: 1.80–2.92) (Table 1). Accordingly, a comparison of the RoseTTAFold models to the same reference structure led to superpositions with a common TM-score of 0.95 and a median RMSD of 1.55 Å (range: 1.53–1.59) (Table 1). Furthermore, the ColabFold and RoseTTAFold models are superimposable with a median TM-score of 0.64 (range: 0.63–0.67) and median RMSD of 3.74 Å (range: 3.43–3.77).

The Protein Data Bank in Europe—Knowledge Base (PDBe-KB) separates the 14 existing experimental structures of PSN1 into two clusters (PDBe-KB: P49768). The two representative entries are 5FN3 and 5FN4 for clusters 1 and 2, respectively. We superposed the predicted ColabFold models to the overlapping region of these structures in order to address the possibility that AlphaFold favors one more than the other, as seen for TREM2. The differences were minimal, and the comparison results are summarized in Supplementary Table S3. Upon careful manual inspection, the definition of two distinct clusters is ambiguous due to their high structural similarity (RMSD < 1.3 Å between 5FN3 and 5FN4).

### 2.3. Pathogenic Variants of PSN-1

Exons 8–9 are critical for the catalytical activity of PSN-1 in the γ-secretase complex, which cleaves many critical proteins for signal transduction, including ErbB4, E-/N-cadherins, and Notch. Exon 8 of the PSN1 gene encodes a specific segment of the protein chain, spanning residues 258–289. Within this region, 18 pathogenic missense mutations have been identified and characterized as per a recent study [29]. Exon 9 only has one missense variant (T291P), and it is known to result in γ-secretase complex with intact structure; it only exerts its effect on impaired proteolysis; therefore, it was not analyzed and discussed in depth. According to the protein disorder database DisProt, this segment belongs at the very beginning of a long-disordered region of the protein, which is located between residues 260 and 378 [30]. The first five mutations involve residues in the transmembrane region of the protein, while the remaining thirteen do not. Affected residues do not show a preference for being hydrophobic or hydrophilic, as they substitute E (*4), V (*3), L (*3), R (*3), A (*2), C (*1), P (*1), and G (*1), with the number in parenthesis indicating how many times a mutation occurs on that type of residue in these pathogenic variants. To be able to hypothesize the likely effect of these pathogenic variants, these missense mutations were mapped onto the three-dimensional structure of the PSN1 protein chain, generated using the AlphaFold algorithm for the canonical PSN1 sequence.

The top structural models (as ranked by pLDDT) for the variants of interest exhibit little deviation from the corresponding wild-type PSN1 model, as observed through structural comparisons. The calculated TM-score and RMSD metrics obtained via the TM-align algorithm for superpositions generated across these variants are displayed in Table 2.

The majority of the superpositions exhibit RMSD values below 1 Å, with the highest RMSD computed for variant R278I at 1.5 Å. None of the variants exhibit a TM-score below 0.97, and there are multiple variants with a TM-score equal to 1. This result clearly suggests that these missense mutations are nearly invisible to the prediction algorithm, and hence do not compromise the structural model.

**Figure 3:**
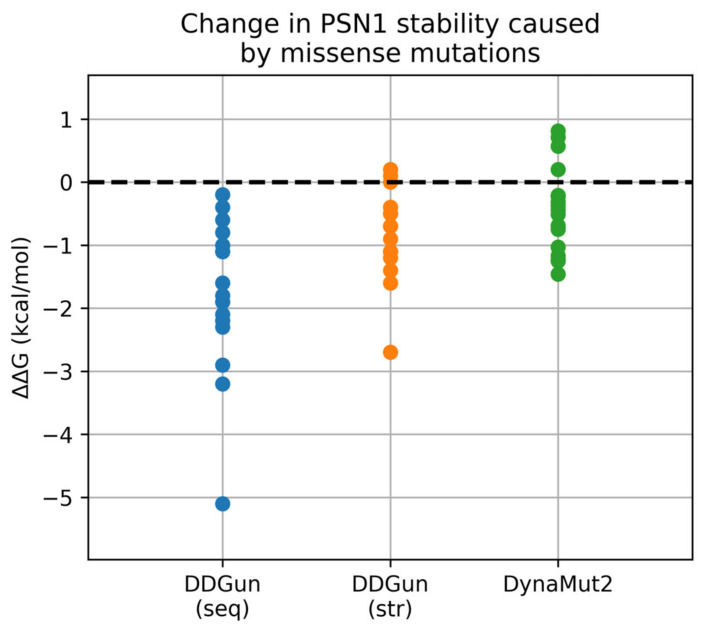
Visualization of the predicted ΔΔG values caused by the 18 missense mutations in PSN1. On the horizontal axis, the three different prediction methods are specified, whereas the vertical axis corresponds to the resulting ΔΔG value in kcal/mol.

To further investigate and address the possible effect of mutations at the protein level, a stability assessment of the variants was performed using the tools DDGun and DynaMut2. For DDGun, both the wild-type sequence and the wild-type model predicted by ColabFold were used as input, along with a file including the 18 mutations. For DynaMut2, only the ColabFold model was supplied along with the mutation file. The predicted change in ΔΔG is calculated in kcal/mol and is shown in Table 2. According to DDGun, very few of the mutations appear to lead to a significant change in stability. Commonly, a threshold is set before assuming that a mutation has a destabilizing/stabilizing effect, since ΔΔG values near 0 usually have no physical meaning. Here, we require the ΔΔG value to be <−1 kcal/mol for a mutation to be considered destabilizing [31]. As shown in Table 2 and visualized in Figure 3, none of the three methods rank any of the mutations with ΔΔG > 1 kcal/mol, and therefore stabilizing. In the DDGun results, all predicted changes in ΔΔG are negative when the sequence is used, suggesting a generally destabilizing effect for most of the mutations. However, only 13 of the 18 mutations result in a ΔΔG < −1 kcal/mol. Upon entering the ColabFold structure as input to the DDGun algorithm, all predicted changes seem to be drawn to more neutral values. Only 7 of the 18 mutations now have a significant change in stability. The stability change predicted by DynaMut2 does not pass the significance threshold for the majority of mutations; only five of them have ΔΔG < −1 kcal/mol. Upon considering a consensus of the three predicted ΔΔG metrics where all three are required to meet the threshold in order to rank a mutation as (de)stabilizing, the destabilizing mutations are V261F, R269G, and R278I, are classified in this category. However, even for these mutations, the predicted change in stability is small.

**Figure 4:**
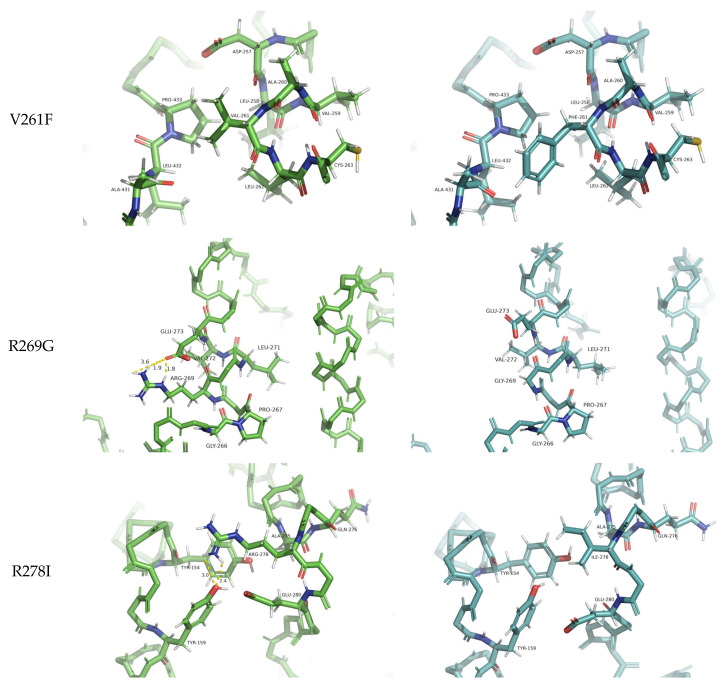
Atomic-level representations of the alterations caused by the three destabilizing variants of PSN-1. The wild-type structure is shown on the left, and the corresponding variant on the right. Side chains are only visible on residues that interact with the main amino acid affected by the mutation. Distances from the main amino acid are displayed only for the polar interactions of the side chains. Hydrogen bonds between atoms of the backbone are not portrayed.

Upon careful inspection of these three destabilizing variants, it is clear that the two arginine mutations result in the largest structural deviations (RMSD: 1.42–1.50 Å), while the valine mutation has no effect on the backbone of the protein (RMSD = 0.24 Å). By considering the physicochemical properties of these residue substitutions, the V261F mutation is the only one preserving the hydrophobic character of the residue. However, valine has a small aliphatic side chain, while phenylalanine has a large aromatic side chain, and for this reason, one can argue that the predicted destabilization must stem from the unfavorably restricted space for such a bulky side chain (Figure 4). Val261 forms hydrophobic contact with Pro433 with optimal side chain-side chain distance. However, the increase in side chain dimension introduced by the Phe261 of the PSN1 variant results in spatial clashing with Leu432. Arginines often stabilize the protein structure (and the binding of protein partners) by ionic interactions and hydrogen bonds. The PSN1 mutations R269G and R278I have taken away the capacity of the residue to form these interaction types. Glycines lack side chains; thus, Gly269 fails to stabilize the structure of the variant by stapling the helix it is located in by three hydrogen bonds (Arg269–Glu273) (Figure 4). The in-depth assessment of the residue-residue interactions of the variant with the other arginine mutation (R278I) loses the ability to stabilize the fold by important non-local residue interactions (2 hydrogen bonds to Tyr159) (Figure 4). Ile278 can form a non-native hydrophobic interaction with Tyr154, but this cannot compensate energetically for the loss of stability stemming from the missing hydrogen bonds and additional loss of native hydrophobic contacts of Tyr154 (Figure 4).

### 2.4. Prediction of Intrinsic Disorder and Binding Residues

**Figure 5:**
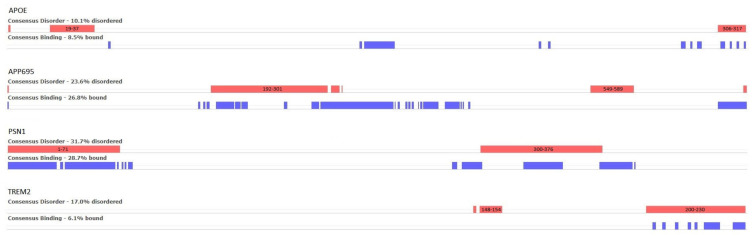
Consensus results from the predictions of disorder (red) and binding residues (blue) generated through the CAID Prediction Portal. The results are filtered using the F1-score optimized threshold. The positions of large, disordered regions on the protein sequence are annotated on the corresponding red bars.

Through the CAID Prediction Portal [32], 32 different methods for the prediction of intrinsic protein disorder (IDRs), as well as 12 different methods for the prediction of binding residues in IDRs, were employed using our AD-related proteins of interest as input. The consensus results are illustrated in Figure 5, where the red bars correspond to disordered regions that are the consensus result of the 31 prediction algorithms, and the blue ones to the consensus prediction of disordered binding residues (9 algorithms).

For APOE, two small, disordered regions are predicted at positions 19–37 and 306–317, of which the first is known in the literature. In the case of APP695, a large, disordered region is predicted between residues 192 and 312 and a smaller one at 549–589. Regarding PSN1, two disordered regions are shown—both are manually curated in DisProt (DP01292). The first is positioned at the beginning of the protein chain at residues 1–71 and has not been experimentally resolved in any existing PDB entries. The second region is at the residue range 300–376 and only appears in 1 out of the 14 PDB structures. Finally, TREM2 is predicted to have a small region of disorder near positions 148–154 and a second one at the end of the chain, in the residue range 200–230. Neither of these two regions has been observed in PDB structures.

**Figure 6:**
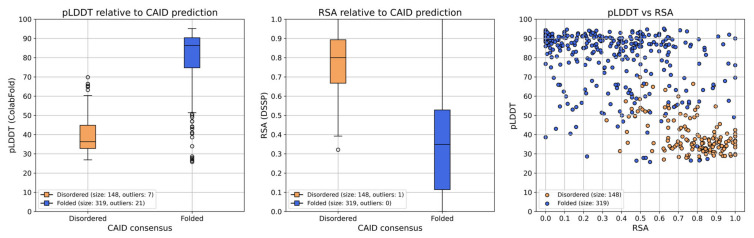
Visualization of the pLDDT and RSA scores of PSN1 residues of the best ColabFold model grouped by the consensus result of the CAID prediction. RSA values were computed using the DSSP algorithm. The two boxplots provide an overview of the spread of the two metrics with regard to their predicted group (disordered or folded). The scatter plot clearly depicts that disordered residues have lower pLDDT scores and are all characterized by high RSA.

Observing the structures and plots generated by ColabFold, and further plotting the pLDDT score and relative solvent accessibility (RSA) of each residue in the ColabFold model of PSN1 for the two CAID prediction result groups (Figure 6) leads to the observation that the regions predicted as disordered are all ranked with very low pLDDT scores and high RSA. Residues in most of these regions have pLDDT scores < 50 in every model of the four proteins of interest, while at the same time, RSA > 0.5 (Figure 6 and Figure S19). The only exception is part of the second disordered region of PSN1 (300–376), where the pLDDT value is also mostly <50, but some of the residues in the region have a score of up to 70 in models 1–4. This indicates conservation imposed by evolutionary pressure to preserve a CDK5 phosphorylation site located at aa. 351–358 (ELM: MOD_CDK_SPxxK_3, ELMI003227) [33]. However, the overall observation that regions predicted as disordered are also ranked with a very low pLDDT value and high RSA agrees with the expectation that these proxies provide decent indications of structural disorder [34].

Upon examination of the disorder predictions, the question arises of whether the disordered binding residues are better conserved than the disordered non-binding ones. To answer this, the pLDDT metric was treated as a means of evolutionary conservation, and the average pLDDT score was calculated for the two groups of disordered binding and disordered non-binding residues. In PSN1, 95 disordered binding residues are predicted. According to the highest ranked ColabFold model, their average pLDDT is 41.5, which is significantly higher (*p* = 0.0013, Mann–Whitney U-test) than that of disordered non-binding residues (total of 53 amino acids) with an average of 36.7. In APP695, there is a total of 58 disordered binding residues, with an average pLDDT of 41.1, and 106 disordered non-binding residues with an average pLDDT of 31.5, which is statistically significantly lower (*p* = 0.0001, Mann–Whitney U-test). Due to the very small number of predicted disordered (binding) residues in APOE and TREM2, no statistical significance could be identified between the corresponding mean pLDDT scores.

These observations in PSN1 and APP695 validate the hypothesis that binding residues located in disordered regions have a higher degree of conservation compared to non-binding residues. Simultaneously, this can be viewed as a weak indication of validation for the given binding predictions. However, to further validate the binding regions within IDRs, we also queried the Eukaryotic Linear Motif (ELM) resource [35] for more annotated binding motifs in all four proteins, similarly as was conducted for PSN1 to identify the CDK5 phosphosite. The ELM search revealed that the putative binding segment on AP695’s C-terminus also partially overlaps with a WW domain recognizing Pin1 prolyl cis/trans isomerase docking site (DOC_WW_Pin1_4, ELMI002308) [36] and fully encompasses a GRB2-SH2 binding motif (LIG_SH2_GRB2like, ELMI003686) [37]. The other putative binding regions of IDRs did not overlap with experimentally verified and manually curated short linear motifs of ELM.

## 3. Discussion

In the present study, four AD-associated proteins were modeled by AlphaFold 2 (AF2) and RoseTTAFold to benchmark the performance of these tools and assess which conformation they capture and how they deal with regions that have not been resolved experimentally. For PSN1, missense variants of the catalytically important exons 8–9 were evaluated to establish whether the structure prediction tools run on constructs with single residue substitutions are sensitive to these changes.

Overall, both AF2 and RoseTTAFold performed very well in this small benchmark. All proteins were modeled with the expected fold with a median TM-score above 0.75 [Table 1]. The most challenging target turned out to be the C-terminal region of APP695 (reference structure: 1TKN), on which both AF2 and RoseTTAFold achieved TM-scores below 0.85 (AF2: 0.77, RoseTTAFold: 0.81). The other template for the N-terminal domain (reference structure 4PWQ) was, on the other hand, modeled with excellent median TM scores of 0.94–0.95 [Table 1]. What the computational models offer as an advantage on top of the well-resolved experimental structures is a full-length 3D model of the protein with a proposed relative orientation and placement of these two building blocks (occasionally connected by unrealistically looking placeholder segments for disordered regions, sometimes decorated by molecular recognition features or disordered binding motifs sampling a conformation with secondary structure (i.e., an alpha-helix). Whether the relative positioning of the two APP695 domains is correct, new experimental structure determination methods (i.e., cryo-electron microscopy suited for large structures) will have to be validated. The unrealistically looking IDRs pose a real challenge for classical structural biology, but are better suited for integrative ensemble modeling efforts [38].

PSN1 as target (PDB: 7D8X) was also proved to be of medium difficulty for AF2 with modeling accuracy measured by the median RMSD of 2.19 Å and TM-score of 0.91, while RoseTTAFold managed to find a solution with RMSD of 1.55 Å and TM-score of 0.95 (Table 1). It is, however, important to note that RoseTTAFold failed to systematically overperform AF2 on all targets; it only generated better models for half of the targets (on the remaining half, AF2 performed a little bit better), as depicted in Table 1. Overall, one can conclude that the two algorithms had comparable precision, and it is generally a good idea to use both tools when building models.

While many regions in the resulting models received a relatively low confidence score—measured by pLDDT (the authors of the method classify 70 > pLDDT > 50 as low confidence, and pLDDT < 50 very low confidence)—as all four AD-associated proteins have IDRs and the low-confidence regions primarily correspond to these, judging the reliability of the models solely based on the pLDDT would be overly simplistic. IDRs are notorious for producing lower pLDDT scores due to their lower sequence conservation and limited structural representation in PDB and conformational diversity and adaptability [39]. It is of note that while the 3D coordinates of these IDRs are not modeled by AF2 based on biophysics principles, the pLDDT of residues in a region was shown to be predictive of intrinsic disorder. Here, we also ran a myriad of disordered predictors through the CAID Prediction Portal and computed consensus results on which we confirmed this predictive power (along with that of RSA of modeled residues), as shown in Figure 5. PLDDT and RSA considered together seem to dissect and make IDR residues cluster (Figure 6). Exceptions of this rule exist, and among them are regions that, due to their high local conservation, obtain higher than expected pLDDT scores—typically, these are short linear motifs (SLiMs) of IDRs that bind partner domains, as exemplified in Section 2 on the CDK5 phosphosite of PSN1.

In addition to predicting IDRs in the target proteins, we also set up consensus predictions for disordered binding sites, which are elementary functional units within these regions. The consensus proposes subregions of IDRs that may carry functional SLiMs. In addition to PSN1, we also validated one of the proposed binding regions of AP695, located on the C-terminus of the protein. It was experimentally shown in earlier studies that a Pin1 prolyl cis/trans isomerase docking site that recognizes WW domains [36] and a GRB2-SH2 binding motif that recognizes SH2 domains [37] was functioning here, and these motifs were already curated in ELM for the protein [35].

Neurodegenerative disorders are characterized by fundamental processes associated with progressive neuronal dysfunction and death, and are typically attributed to accumulations of specific proteins, which are localized in β-amyloid-containing formations in the case of AD, α-synuclein in Parkinson’s Disease and various ubiquitinated proteins, like TDP-43, in Amyotrophic Lateral Sclerosis [40]. These aggregates show a characteristic cellular and neuroanatomical distribution in neurons or glial cells. Examples of protein aggregation within neurons include tau in neurofibrillary tangles (NFTs) or Pick bodies, α-synuclein in Lewy bodies, and TDP-43 in neuronal cytoplasmic and neuronal intranuclear inclusions [41]. In these abnormal protein aggregates, intrinsic neuronal proteins display secondary structures enriched in β-sheets.

Amyloids are insoluble fibrous proteins with specific structural features, including a secondary structure rich in β-sheets. The protein disorders of almost all common neurodegenerative diseases have amyloid features [42]. Amyloid morphological variation is specific to each amyloid type or species, disease stage, and neuroanatomical location. However, Aβ is a defining feature of Alzheimer’s disease; Aβ amyloid deposits are found as a comorbid feature of many neurodegenerative disorders in the elderly, especially in individuals who carry the major genetic risk factor for AD, apolipoprotein E4 [43].

APP is processed through amyloidogenic and non-amyloidogenic pathways. It is cleaved either by α-secretase (non-amyloidogenic pathway) or β-secretase -BACE1- (amyloidogenic pathway), generating α- or β-C-terminal fragments, respectively, which are attached to the membrane. Specifically, digestion of APP by α-secretase releases sAPPa from the cell surface and leaves a C-terminal fragment of 83 amino acids. In the alternative pathway, APP is digested by β-secretase, releasing sAPPβ and leaving a C-terminal fragment of 99 amino acids. Amyloidogenic processing of APP involves sequential cleavages by β- and γ-secretase at the N and C termini of Aβ, respectively, with β-secretase cleaving APP at position Asp1 or Glu11 of the Aβ sequence. Furthermore, cleavage of C99 by γ-secretase liberates an APP Intracellular Domain (AICD) that can translocate to the nucleus, contributing to the regulation of gene expression, including the induction of apoptotic genes [44]. The γ-secretase complex consists of four protein subunits: presenilin (PSN), presenilin enhancer (PEN), APH, and nicastrin. There are many isoforms of PSN (PSN1/PSN2), while mutations consistently reduce the productivity of γ-secretase and release of longer Aβ peptides [45].

In our analysis, PSN1 was further explored in terms of sequence variation by evaluating the structural and energetic consequence of pathogenic missense mutations on the catalytically important exons of the protein (exons 8–9). The overwhelming majority of the mutations were located in exon 8 (18 out of the total 19). Interestingly, most of these mutations were found to substitute either charged or hydrophobic residues. Both structural assessment (by AF2) and energetic elucidation of the effect on protein stability (by DDGun and DynaMut2) highlighted that most of these single amino acid substitutions had negligible effect in these regards. Theoretically, this could mean that the majority of the pathogenic variations only compromise the substrate binding and the underlying catalytic efficiency. On the other hand, we also found that three missense mutations had a destabilizing effect on PSN1, classified as the consensus of the two predictors with ddG < −1 kcal/mol. Using consensus prediction, over- and underestimation of the destabilization effects measured on the variants can be mitigated, especially in light of our finding that DynaMut seems to systematically score the effects less serious compared to DDGun. Furthermore, we found that DDGun predictions purely on the sequence have overestimated the effects of the mutations without the structural context of this amino acid substitution. This underlines the importance of using structural information whenever possible, and in the era of AF2 and RoseTTAFold, generating structural models is easier than ever. Unless the mutations are located within regions with very low local model confidence (e.g., measured by pLDDT), using a reliable model can yield important information on top of the sequence information.

Among the three mutations (V261F, R269G, R289I) exhibiting the lowest predicted destabilization ddG scores (−1.23, −1.25, −1.17 by DynaMut2 and −1.1, −1.4, −1.1 by structure-aware DDGun, respectively) [Table 2], diverse putative destabilization mechanisms were found. We hypothesize that V261F slightly destabilizes the protein structure by introducing an oversized aromatic side chain in place of a small aliphatic side chain in a well-pack environment. While for the R269G and R289I mutations, we also found structural consequences manifesting in RMSD values of the superpositions with the wild-type structure (RMSDs of 1.42 and 1.50 Å) accompanying the predicted destabilization free energy differences. This is noteworthy considering the fact that the two arginines are located at the beginning of an IDR annotated for PSN1 (DisProt ID: DP01292). The proposed mechanism of destabilization for the R269G mutation is the loss of 3 local helix-stapling hydrogen bonds from the arginine to the residue in the next helical turn (Glu273), and as glycine lacks side chain, these side chain-side chain hydrogen bonds are lost. For R278I, we predicted that non-local hydrogen bonds are lost that normally point to distant structural segments and thus stabilize the fold. In the wild-type structure, Arg278 forms two hydrogen bonds with Tyr159, while the Ile278 variant can only form a hydrophobic interaction with Tyr154. However, this way, Tyr154 fails to form 4 other hydrophobic contacts (Leu150, Leu271, Ala275, Phe283) present in the wild-type model, which explains the predicted destabilization.

## 4. Materials and Methods

### 4.1. Selection of Wild Type Proteins and Mutated Variants

Four proteins related to Alzheimer’s disease pathology were selected and examined. Namely, Presenilin-1 (PSN1), Apolipoprotein E (APOE), Isoform APP695 of Amyloid-beta precursor protein (APP695), and Triggering receptor expressed on myeloid cells 2 (TREM2). Their sequences were retrieved from UniProt using the accession codes P49768 for PSN1, P02649 for APOE, P05067-4 for APP695, and Q9NZC2 for TREM2. For each of the four proteins, reference structures were selected and fetched from the PDB using the corresponding chains from the entries with Entry ID: 7D8X (chain B) for PSN1 [46], 7FCR for APOE [47], 4PQD (chain A) and 1TKN (model 1) for APP695 [48,49], and 5UD8 (chain B) and 5ELI (chain A) for TREM2 [50,51]. The primary criterion for reference structure selection was the overall resolution of the resolved structure. In exon 8 of PSN1, there is a total of 42 missense mutations. Out of those, 18 have been ranked as pathogenic according to a recent re-evaluation study of genetic variants implicated in AD [29]. These pathogenic mutated variants were selected for further examination because these exons carry a region of importance for the cleavage of target proteins.

### 4.2. ColabFold Parameters and Execution

For the computational analysis of protein structures, the open-source software ColabFold [9] was employed (versions 1.3.0 and 1.5.2). The notebook “ColabFold: AlphaFold2 using MMseqs2” was used through the Google Collaboratory platform to run AlphaFold with the following parameters:

use_templates = false, use_amber = true, msa_mode = “MMseqs2 (UniRef + Environmental)”, model_type = “AlphaFold2-ptm”, num_models = 5, model_order = [1, 2, 3, 4, 5], num_recycles = 6, rank_by = “plddt,” max_msa = null, pair_mode = “unpaired + paired”.

The wild-type sequences of the four proteins of interest (PSN1, APOE, APP695, and TREM2), as well as the 18 selected pathogenic variants of PSN1, were input to ColabFold for structure prediction using the specified parameters. No templates were used in the predictions. With the specified parameters, the AlphaFold algorithm generates five different structural models for each individual input. The models are ranked from best to worst based on the overall predicted Local Distance Difference Test (pLDDT), AlphaFold’s per-residue confidence metric. Along with the structural models, the Predicted Aligned Error (PAE) of each model is visualized on a matrix. This metric implies a measure of confidence in the relative positions of residue pairs. All five of the models produced for each protein were used in our analyses. Additionally, all the structural models have undergone relaxation in the Amber99sb force field [52] as part of the AlphaFold framework. For the mutated variants of PSN1, only the model with the highest pLDDT was relaxed using Amber. To greatly improve the accuracy of our outcomes, the number of recycles was doubled to 6 instead of the default three cycles.

Furthermore, the RoseTTAFold algorithm was executed on the four proteins of interest so that comparisons could be made among the results of the two deep learning methods, including the experimental PDB structures. To run the RoseTTAFold algorithm, the Robetta web server was used (https://robetta.bakerlab.org accessed on 19 July 2023) [15].

### 4.3. Structure Evaluation Metrics

For each protein, all five of the predicted models were compared to the corresponding reference structures fetched from the PDB. Similarly, all five models predicted by RoseTTAFold were used for comparison against the reference structures. The TM-score and RMSD metrics of the C_a_ atoms were computed for each predicted-reference structure pair using the TM-align [26] algorithm on the PyMOL software (The PyMOL Molecular Graphics System, Version 2.5 Schrödinger, LLC). In the current case study, the reference structures match only with specific domains of the full region of the protein. Thus, most comparisons are limited to these well-characterized domains. A brief assessment of the most significant deviations between the in silico and experimental models was also performed and discussed in detail.

In exon 8 of PSN1, 18 pathogenic mutations have been identified as pathogenic for AD; another one is identified in exon 9 [29]. ColabFold was executed for these 19 pathogenic variants, and the highest-ranked model of each variant was superposed to the ColabFold structure of the wild-type PSN1 sequence produced in the previous step. The TM-score and RMSD metrics were computed just as in the wild-type case.

### 4.4. Additional Prediction Tools

The DDGun [53] and DynaMut2 [54] predictors were employed to evaluate the impact of mutations on the thermodynamic stability of the 18 pathogenic PSN1 variants from exon 8. In essence, these tools estimate the change in protein stability upon the introduction of one or multiple missense mutations. To accomplish this, both the amino acid sequence and the top model generated by ColabFold were used as inputs to DDGun, yielding separate results for each input, whereas the DynaMut2 input only consisted of the ColabFold model. To decide on the effect each mutation has on protein stability, the consensus of these three methods was taken into consideration. The criteria for distinguishing a mutation’s effect as destabilizing was a ΔΔG value < −1 kcal/mol, and >1 kcal/mol for a stabilizing one.

The Protein Interactions Calculator (PIC) [55] was employed to calculate intraprotein interactions (hydrophobic interactions, hydrogen bonds, ionic bonds, etc.) occurring among different atoms in the wild-type model of PSN1 and the mutated structures of the destabilizing variants. The result was filtered for the mutated amino acids, and a thorough examination was performed using PyMOL to evaluate the atomic distances of existing non-covalent bonds. Changes in biochemical properties were addressed to enhance our understanding of the effect of these mutations.

To strengthen the analysis, various prediction tools were employed through the CAID Prediction Portal [32] to evaluate the existence of disordered regions and binding residues. For regions of intrinsic disorder, 31 different methods were utilized, and 9 for the identification of binding residues. In the current analysis, the “AlphaFold-disorder“ prediction (which fits both categories) was excluded in order to compare the consensus prediction to the ColabFold structures we generated using six recycling cycles instead of the default 3. The exhaustive list of the methods used may be found in the documentation of the CAID Prediction Portal. Upon execution of the algorithms, the “F1s optimized” binary score threshold (described in the CAID documentation) was used [56]. The consensus results of the predictions were examined and compared to the pLDDT plots generated by ColabFold. The hypothesis that binding residues in disordered regions are more conserved than non-binding residues in the same regions was examined using the pLDDT score as a measure of conservation.

To calculate relative solvent accessibility (RSA) for residues in the models, the DSSP algorithm was run on the structures, implemented in BioPython (Bio.PDB) [57] with accessible surface area calculations following the protocol of Sander and Rost [58].

## 5. Conclusions

Our findings suggest that potent conformations provided by ColabFold in the set of specific proteins involved in AD could be accurately modeled at a molecular level in their wild-type form. Compared regions were correctly folded, and the estimated metrics were almost exclusively in the “high accuracy prediction” range. Most of the deviations depicted between predicted and reference models were mainly located around residues that form loops. Such deviations are acceptable since IDRs and loops are allowed to have structural deviations due to their conformational heterogeneity. The accuracy of ColabFold and RoseTTAFold are on the same level of performance with very slight differences, so it appears that both deep learning algorithms are highly accurate on folded domains—at least in the scope of the current case study. Furthermore, AlphaFold’s pLDDT metric proves itself as a good indicator of intrinsic disorder, as the CAID-predicted IDRs are all ranked with very low pLDDT scores, and since some of the regions have been either annotated in DisProt or verified to be disordered in earlier studies. Moreover, the residues in IDRs that are predicted to bind tend to be more conserved—and thus result in higher pLDDT—than those that do not.

Our study also demonstrated that only a few residues with sufficiently strong destabilization effects can exert detectable structural (backbone) deviation from the wild-type model. In our dataset, only 2 out of 19 mutations (R269G and R278I) of PSN1 resulted in structural perturbations with backbone RMSD > 1.4 Å and, at the same time, predicted free energy differences ddG < −1.0 kcal/mol. Moreover, we showed that these effects can be a consequence of diverse destabilization mechanisms. A substantial limitation of the present study lies in the fact that only a small set of proteins was included in the analysis; furthermore, consideration must be given regarding the accuracy of used prediction algorithms. Notwithstanding, the provided insights are encouraging for future more systematic analyses on a broader set of AD-related proteins, and may help design new directed research focusing on the validation of domain-domain orientations, putative molecular recognition features, or proposed structural effect of the destabilizing mutations in one of the four proteins.

## Figures and Tables

**Table 1:** Comparison metrics (TM-score and RMSD) for PSN1, APOE, APP695, and TREM2 were calculated by superposing the structural models provided by ColabFold and RoseTTAFold against the reference structures fetched from PDB. Superpositions and calculations were made using the TM-align algorithm on the PyMOL software (open source 2.5). The median TM-score and median RMSD of the five predicted models are reported for each prediction-reference pair. The range of values among the five models is given in square brackets in the format: [lowest–highest].

Protein (UniProt ID)	Reference (PDB)	Region Compared	TM-Score (AlphaFold2)	RMSD (AlphaFold2)	TM-Score (RoseTTAFold)	RMSD (RoseTTAFold)
PSN1 (P49768)	7D8X	76−289 and 377−467	0.91 [0.88−0.94]	2.19 Å [1.80−2.92]	0.95 [0.95]	1.55 Å [1.53−1.59]
APOE (P02649)	7FCR	41−181	0.96 [0.95−0.97]	1.09 Å [0.86−1.44]	0.95 [0.94−0.95]	1.11 Å [1.07−1.28]
APP695 (P05067-4)	4PWQ	28−189	0.95 [0.94−0.95]	1.31 Å [1.27−1.52]	0.94 [0.93−0.94]	1.54 Å [1.38−1.64]
	1TKN	460−569	0.77 [0.77−0.78]	2.58 Å [2.52−2.69]	0.81 [0.76−0.82]	2.35 Å [2.23−2.71]
TREM2 (Q9NZC2)	5UD8	19−130	0.94 [0.94]	1.55 Å [1.54−1.56]	0.91 [0.90−0.92]	1.83 Å [1.79−1.90]
	5ELI	19−133	0.98 [0.97−0.98]	0.60 Å [0.57−0.66]	0.95 [0.94−0.95]	0.98 Å [0.92−1.01]

**Table 2:** Table of PSN1 variants. The first column describes the mutation. The second and third columns correspond to RMSD and TM-score comparison metrics, respectively. These metrics were calculated via the TM-align algorithm by superposing the top model output by ColabFold for each of the selected mutated variants of PSN1 and the wild-type PSN1 structure generated in 2.3. The change in ΔΔG was predicted using the tools DDGun and DynaMut2 through their web server implementations. DDGun was executed using two different inputs, the protein sequence (ΔΔG seq) and ColabFold structure (ΔΔG str). For DynaMut2, only the ColabFold structure was provided as input. The last three columns summarize the protein stability change upon mutation. The unit for ΔΔG values is kcal/mol. Bold ΔΔG values are outside the range (−1, 1) independent of the choice of predictor, and are therefore considered destabilizing if ΔΔG < −1 kcal/mol.

Mutation	RMSD	TM-Score	ΔΔG Seq (DDGun)	ΔΔG Str (DDGun)	ΔΔG (DynaMut2)
A260V	0.14 Å	1.00	−0.6	0.2	−0.75
**V261F**	0.24 Å	1.00	**−2.9**	**−1.1**	**−1.23**
L262F	0.15 Å	1.00	−1.9	−0.5	−0.69
C263R	0.19 Å	1.00	−2.1	−1.2	0.71
P264L	0.17 Å	1.00	−2.2	−0.4	−0.21
G266S	0.37 Å	1.00	−1.8	−1.1	−0.21
**R269G**	1.42 Å	0.97	**−2.3**	**−1.4**	**−1.25**
V272A	0.16 Å	1.00	−3.2	−1.6	−0.44
V272D	0.20 Å	1.00	−5.1	−2.7	−0.51
E273A	0.91 Å	0.98	−0.2	0.0	−0.31
A275V	1.10 Å	0.98	−0.4	0.1	−1.03
**R278I**	1.50 Å	0.97	**−2.2**	**−1.1**	**−1.17**
R278K	0.48 Å	1.00	−1.0	−0.7	0.57
E280A	0.18 Å	1.00	−1.1	−0.5	−0.37
E280G	0.37 Å	1.00	−1.6	−0.9	−1.46
E280K	1.26 Å	0.97	−0.8	−0.4	−0.23
L282R	1.35 Å	0.97	−1.1	−0.7	0.81
L286V	0.14 Å	1.00	−1.9	−0.7	0.20
T291P	0.90 Å	0.99	−1.0	−0.8	−0.36

## Data Availability

Not applicable.

## Supplementary Materials

The following supporting information can be downloaded at https://www.mdpi.com/article/10.3390/ijms241713543/s1.

## Abbreviations

Aβ: amyloid β peptide, AD: Alzheimer’s Disease, AF2: AlphaFold2, AI: Artificial Intelligence, APOE: apolipoprotein E, APP: Amyloid-β precursor protein, CAID: Critical Assessment of protein Intrinsic Disorder prediction, CASP: Critical Assessment of Protein Structure Prediction, ELM: Eukaryotic Linear Motif, IDR: Intrinsically Disordered Region, ML: Machine Learning, MSAs: Multiple Sequence Alignments, MMseqs2: Many-against-Many sequence searching, NFT: neurofibrillary tangles, PAE: Predicted Aligned Error, PDB: Protein Data Bank, pLDDT: predicted Local Distance Difference Test, PSN1: presenilin 1, RMSD: Root-Mean-Square Deviation, RSA: relative solvent accessibility, SLiM: short linear motif, TREM2: Triggering receptor expressed on myeloid cells 2.
