# Supplementary material for: In Silico Structural Analysis Exploring Conformational Folding of Protein Variants in Alzheimer’s Disease
Source: Int J Mol Sci. 2023 Aug 31;24(17):13543. doi: 10.3390/ijms241713543 (PMC10487466; doi:10.3390/ijms241713543)

Supplementary Material

In Silico Structural Analysis Exploring Conformational Folding of Protein Variants in Alzheimer's Disease

Evangelos Efraimidis, Marios G. Krokidis, Themis P. Exarchos, Tamas Lazar and Panagiotis Vlamos*

*Correspondence: vlamos@ionio.gr

Table S1. Sample config file from ColabFold run

ColabFold run


```
{
  "num_queries": 1,
  "use_templates": false,
  "use_amber": true,
  "msa_mode": "MMseqs2 (UniRef+Environmental)",
  "model_type": "AlphaFold2-ptm",
  "num_models": 5,
  "num_recycles": 6,
  "num_ensemble": 1,
  "model_order": [1, 2, 3, 4, 5],
  "keep_existing_results": false,
  "rank_by": "plddt",
  "max_msa": null,
  "pair_mode": "unpaired+paired",
  "host_url": "https://api.colabfold.com",
  "stop_at_score": 100.0,
  "stop_at_score_below": 0,
  "recompile_padding": 1.0,
  "recompile_all_models": false,
  "commit": "###########",
  "is_training": false,
  "version": "1.3.0"
}
```

Table S2. Sequence of PSN1 including 19 pathogenic missense mutations marked in red.

MTELPAPLSYFQNAQMSEDNHLSNTVRSQNDNRERQEHNDRRSLGHPEPLSNGRPQGN
SRQVVEQDEEEDEELTLKYGAKHVIMLFVPVTLCMVVVVATIKSVSFYTRKDGQLIYTPFT
EDTETVGQRALHSILNAAIMISVIVVMTILLVVLYKYRCYKVIHAWLIISSLLLLFFFSFIYLG
EVFKTYNVAVDYITVALLIWNFGVVGMISIHWKGPLRLQQAYLIMISALMALVFIKYLPE
WTAWLILAVISVYDLVVFFRLKSPLGMLAATVQEINKTRFPAVIYSSPMVWLVNMAEGDP
EAQRRVSKNSKYNAESTERESQDTVAENDDGGFSEEWEAQRDSHLGPHRSTPESRAAVQ
ELSSSILAGEDPEERGVKLGLGDFIFYSVLVGKASATASGDWNTTIACFVAILIGLCLTLLLL
AIFKKALPALPISITFGLVFYFATDYLVQPFMDQLAFHQFYI

Table S3. Comparison metrics calculated upon superposition of the wild-type ColabFold structure of PSN1 against the two cluster representative structures (5FN3 and 5FN4) as annotated in PDBe-KB. Scores in green represent the better value among the two entries.

	5FN3		5FN4	
	TM-score	RMSD	TM-score	RMSD
AF rank 1	0.934	1.67	0.922	1.82
AF rank 2	0.933	1.55	0.927	1.71
AF rank 3	0.938	1.59	0.929	1.75
AF rank 4	0.939	1.58	0.928	1.73
AF rank 5	0.929	1.64	0.932	1.66

Figure S1. Structural superposition of the PSN1 model provided by ColabFold in blue against the reference structure 7D8X (chain B) in green.

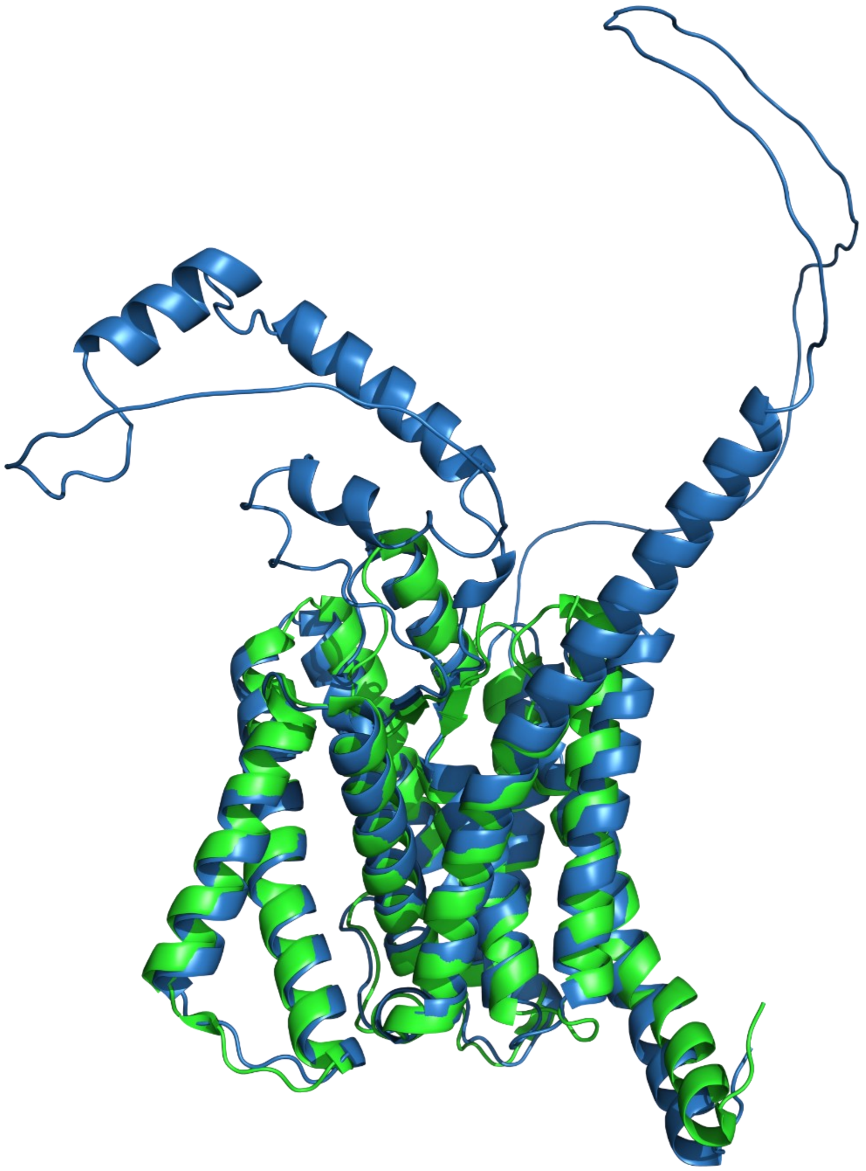

Figure S2. Structural superposition of the APP695 model provided by ColabFold in blue against the reference structures 4PWQ (chain A) in green and 1TKN in purple.

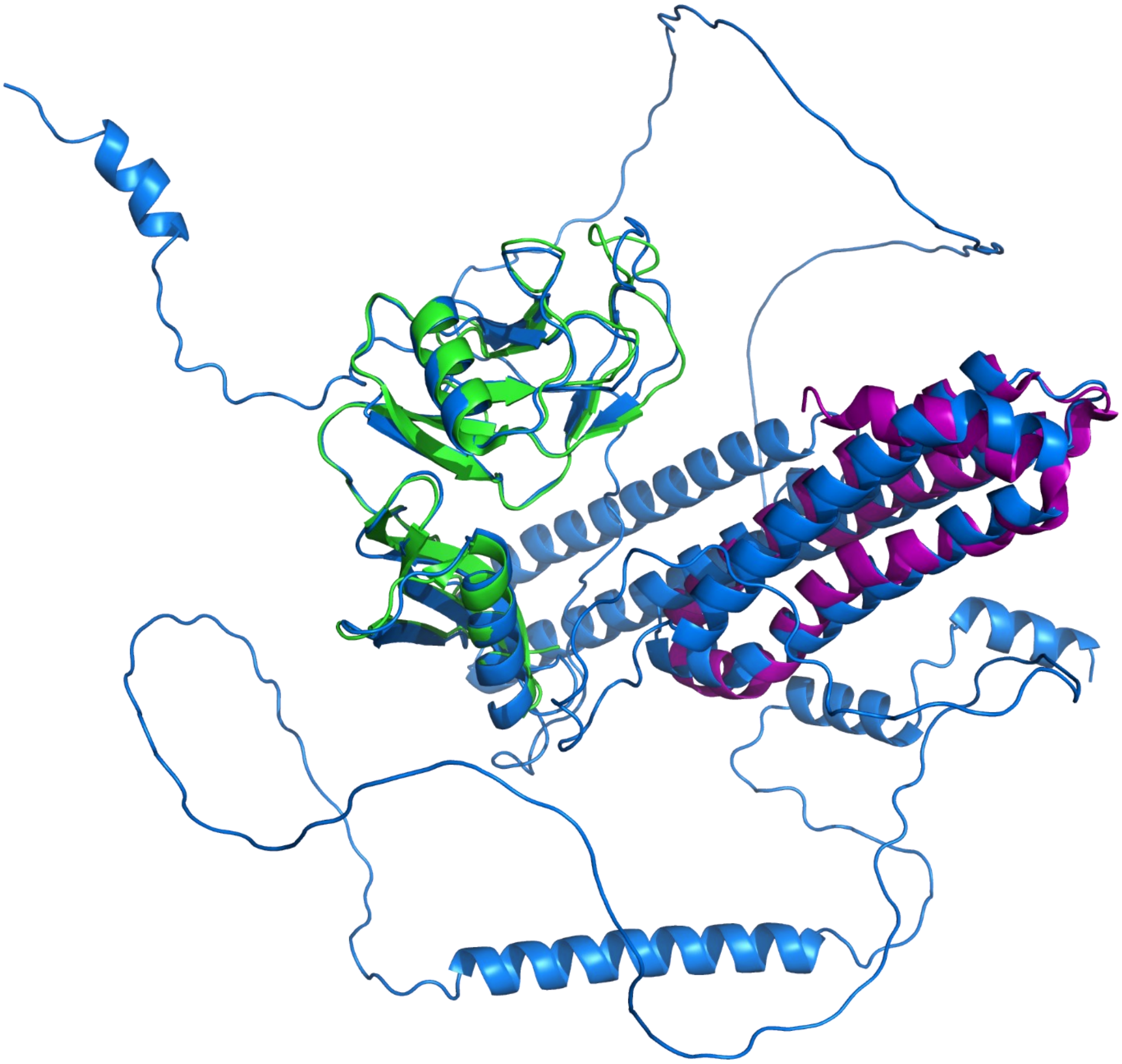

Figure S3. Structural superposition of the APOE model provided by ColabFold in blue against the reference structure 7FCR in green.

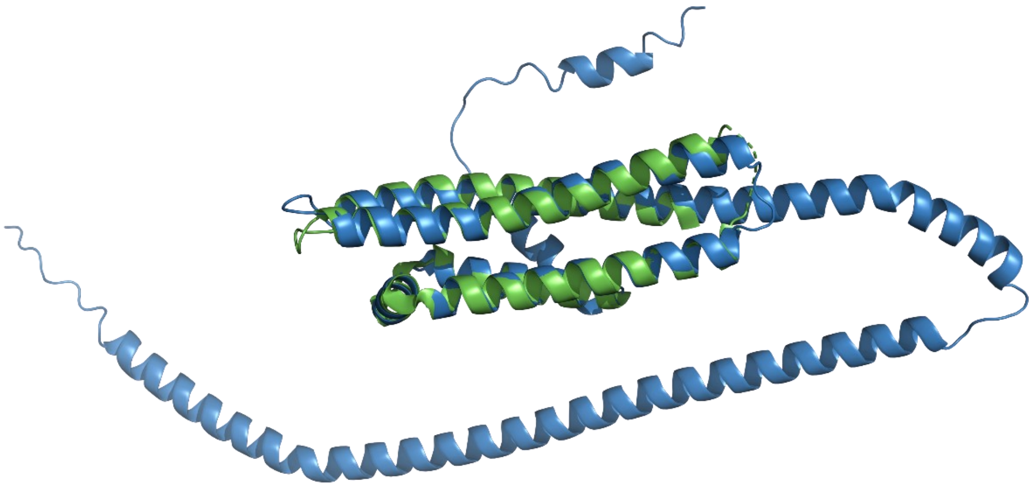

Figure S4. Structural superposition of the TREM2 model provided by ColabFold in blue against the reference structure 5UD8 (chain B) in green.

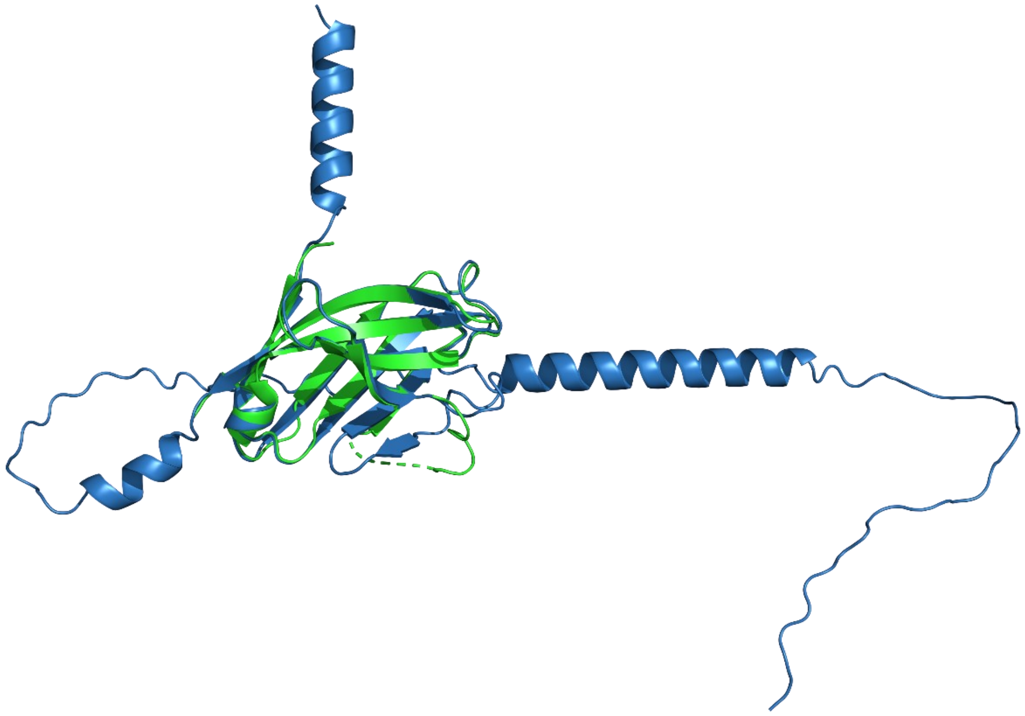

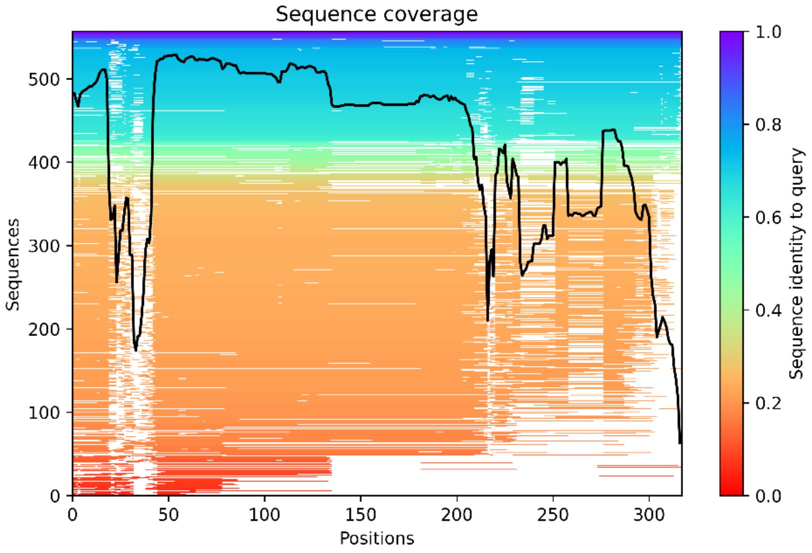

Figure S5. Sequence coverage in the APOE prediction.

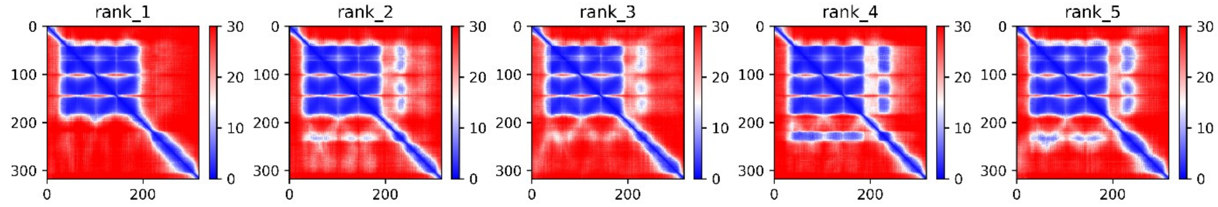

Figure S6. PAE matrices of all five predicted models of APOE, ranked from the best to the worst according to pLDDT.

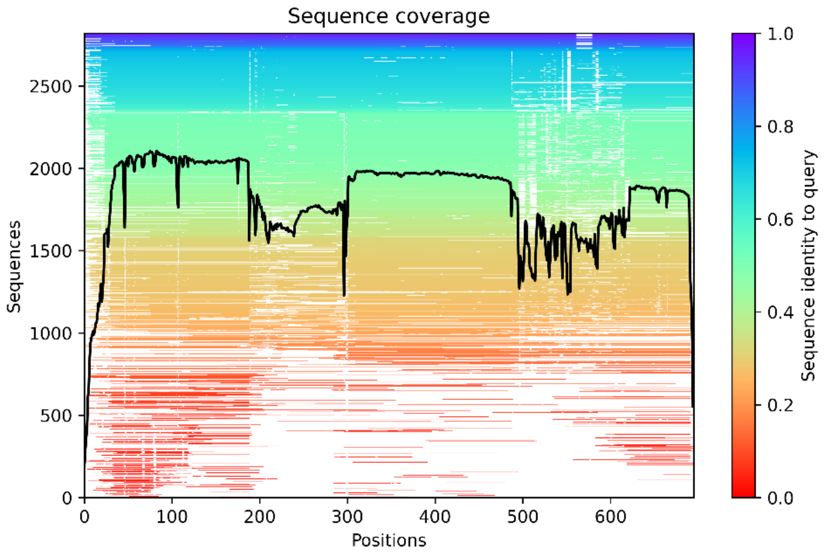

Figure S7. Sequence coverage in the APP695 prediction.

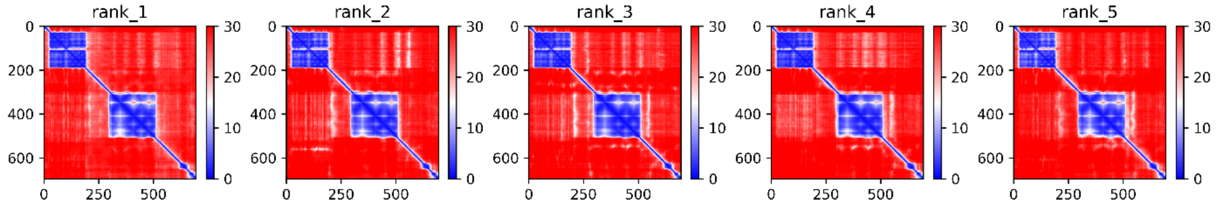

Figure S8. PAE matrices of all five predicted models of APP695, ranked from the best to the worst according to pLDDT.

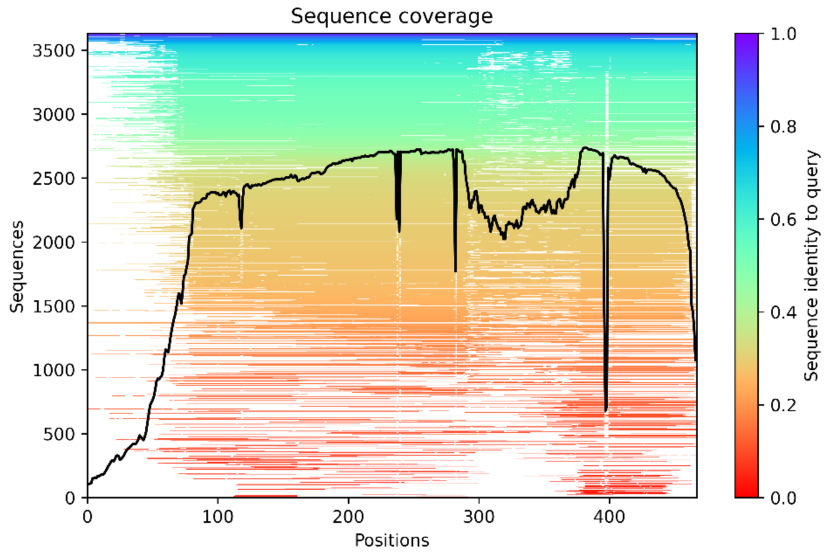

Figure S9. Sequence coverage in the PSN1 prediction.

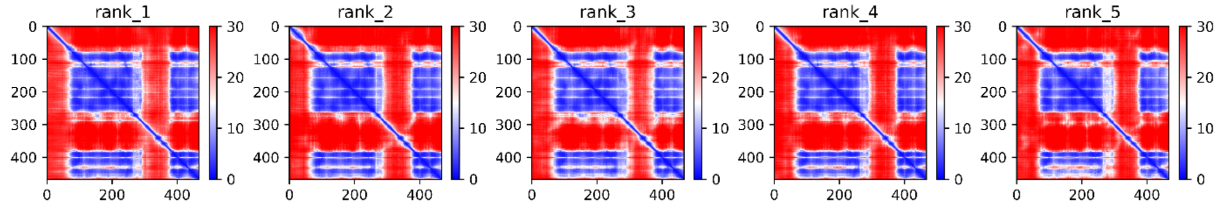

Figure S10. PAE matrices of all five predicted models of PSN1, ranked from the best to the worst according to pLDDT.

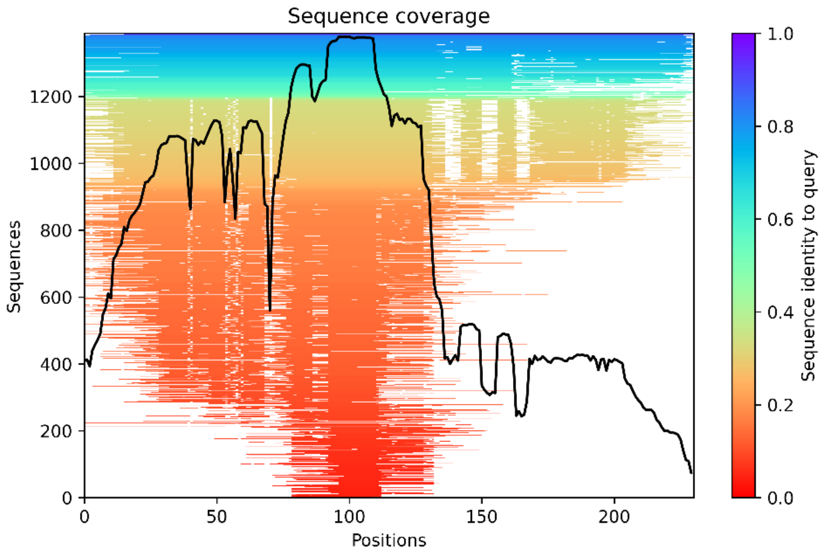

Figure S11. Sequence coverage in the TREM2 prediction.

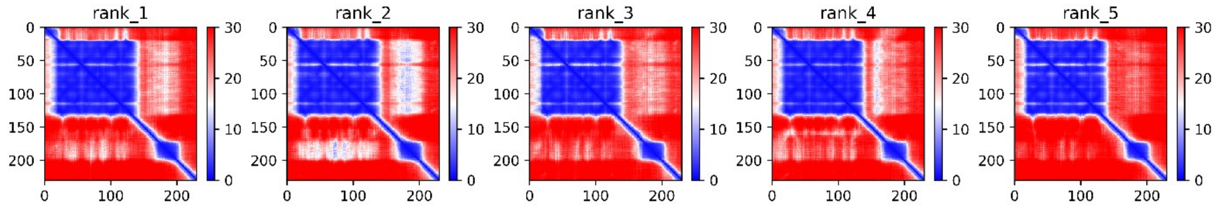

Figure S12. PAE matrices of all five predicted models of TREM2, ranked from the best to the worst according to pLDDT.

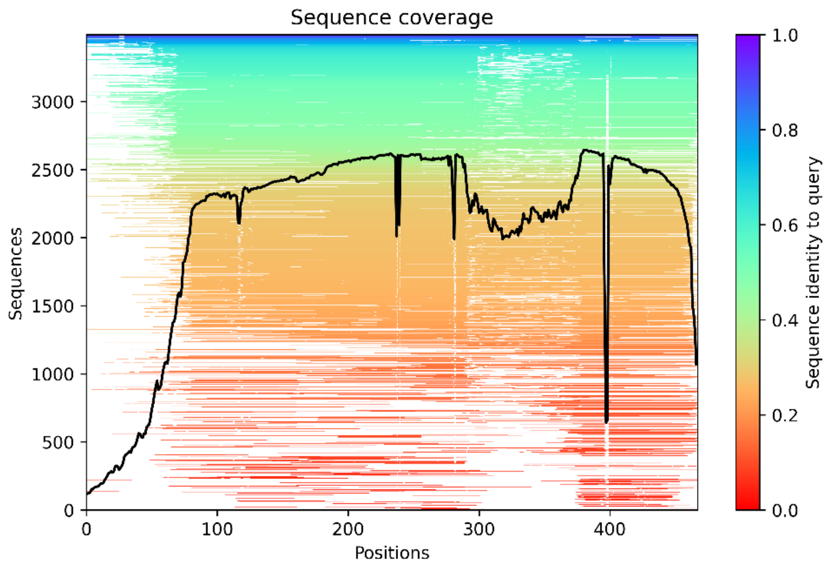

Figure S13. Sequence coverage in the prediction of the PSN1 sequence carrying 18 pathogenic missense mutations found on exon 8.

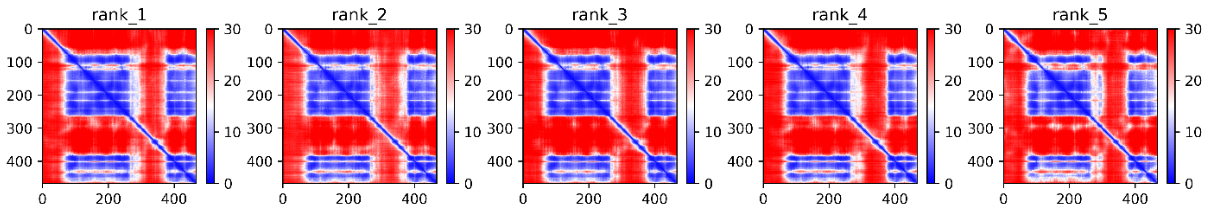

Figure S14. PAE matrices of all five predicted models of the PSN1 sequence carrying 18 pathogenic missense mutations found on exon 8, ranked from the best to the worst according to pLDDT.

Plots from RoseTTAFold runs

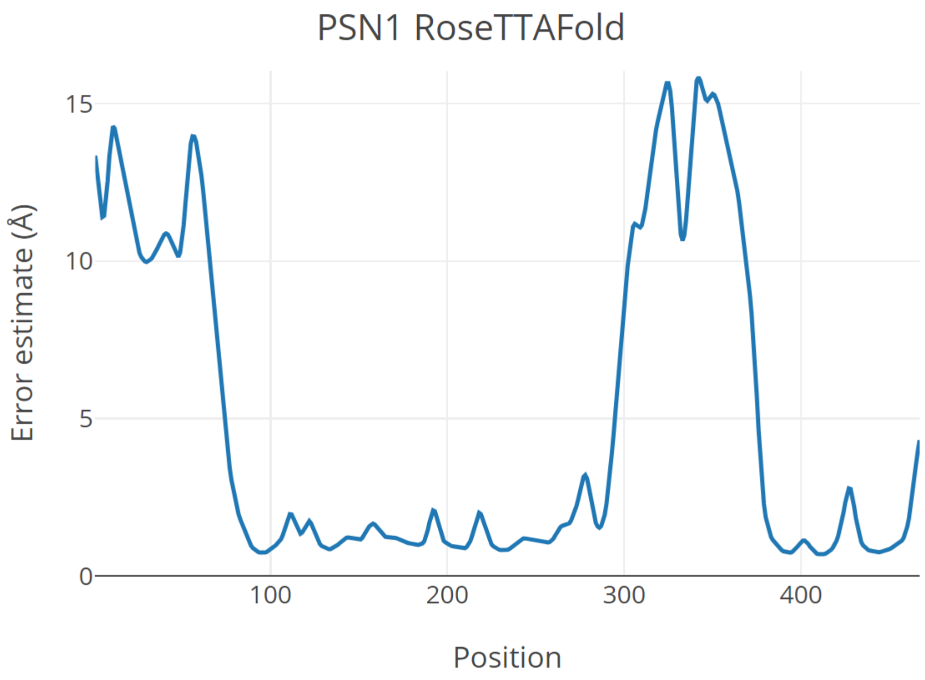

Figure S15. Estimate error plot for the first PSN1 model generated by RoseTTAFold.

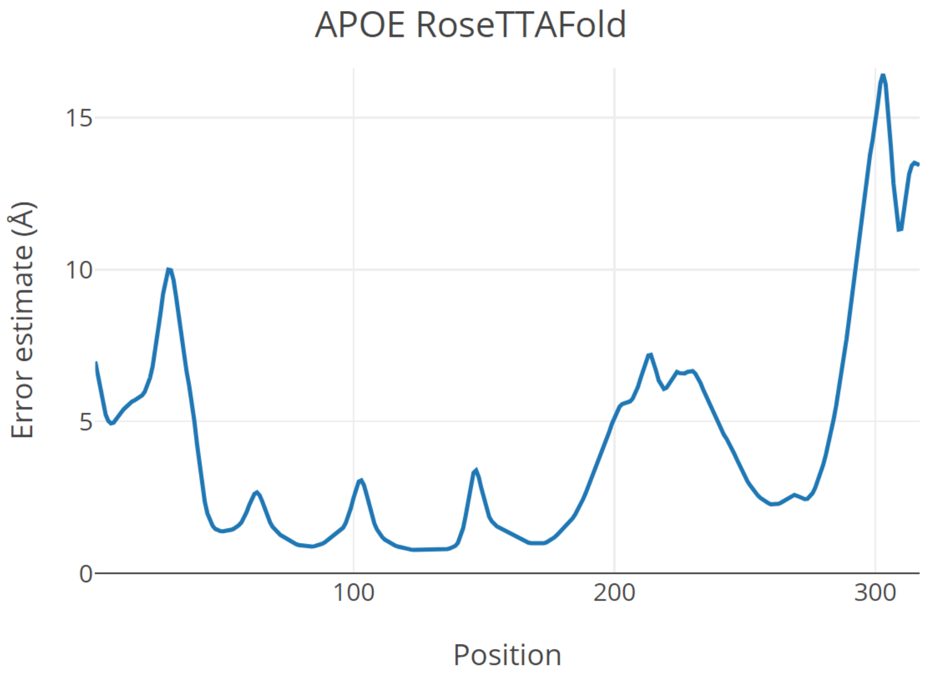

Figure S16. Estimate error plot for the first APOE model generated by RoseTTAFold.

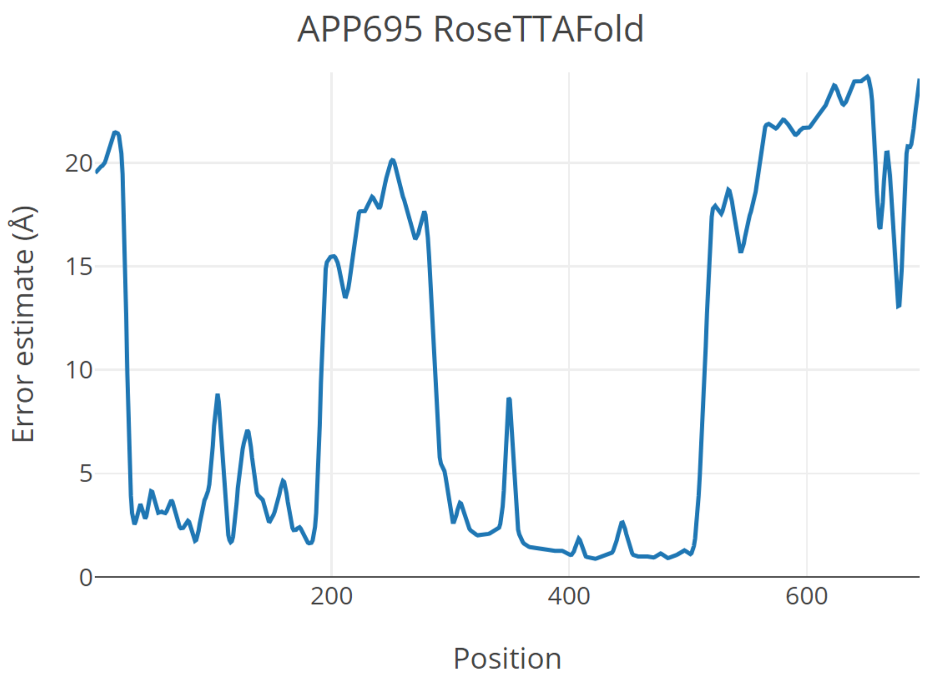

Figure S17. Estimate error plot for the first APP695 model generated by RoseTTAFold.

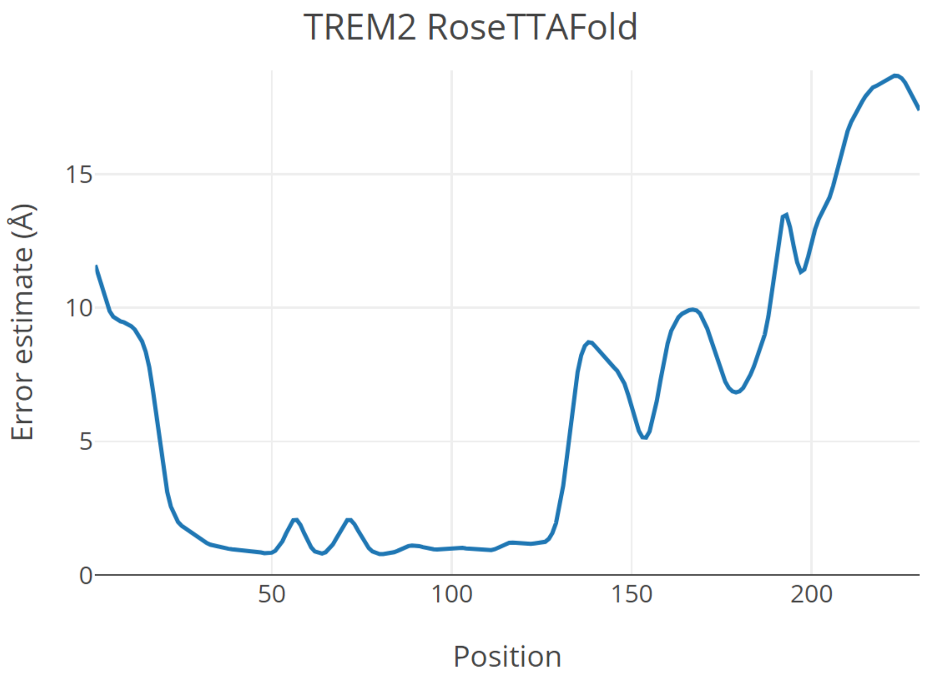

Figure S18. Estimate error plot for the first TREM2 model generated by RoseTTAFold.

Figure S19. Visualization of the pLDDT and RSA scores of residues of the best ColabFold model of the four proteins grouped by the consensus result of the CAID prediction. RSA values were computed using the DSSP algorithm.

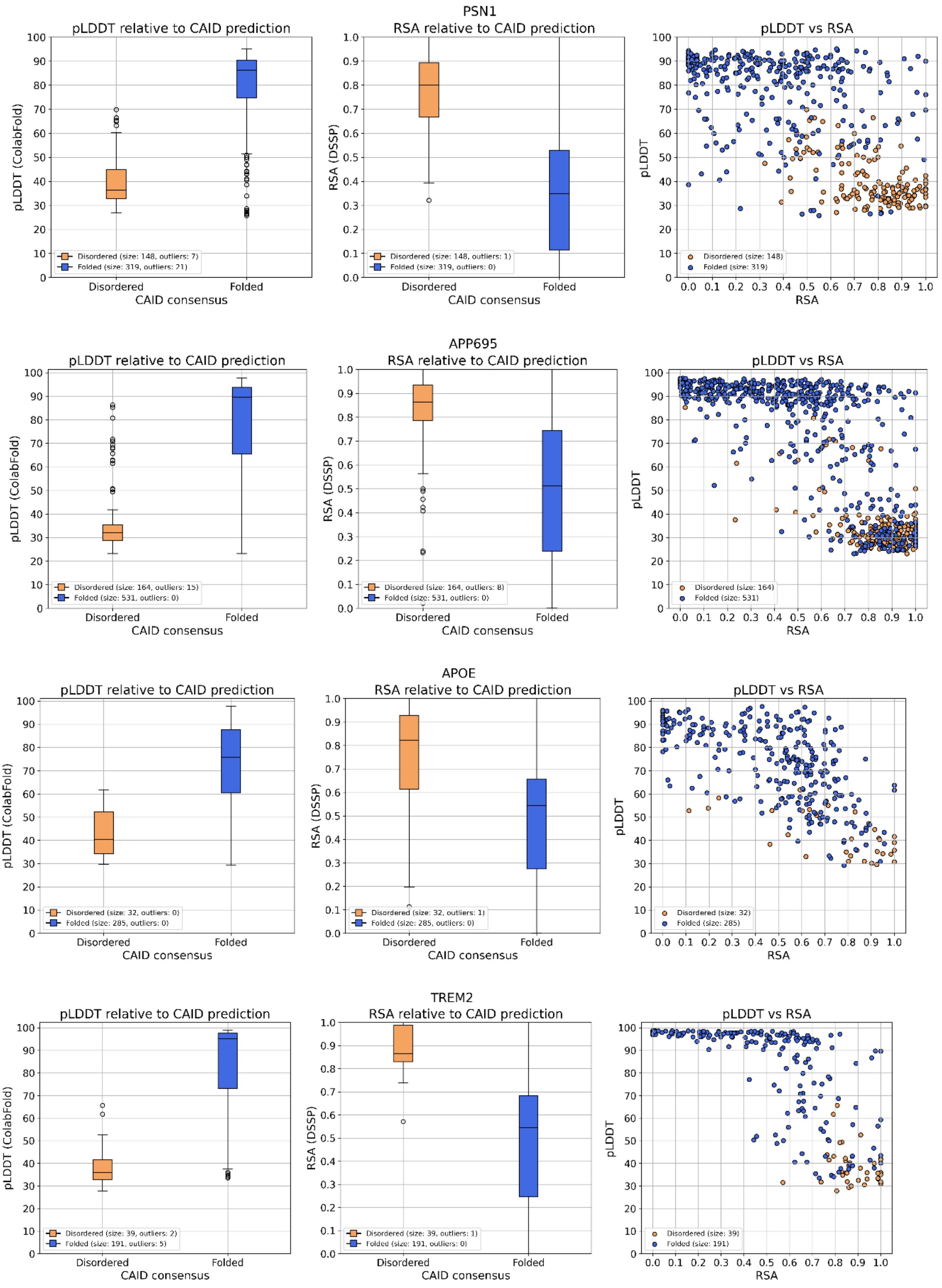

Supplement: Supplementary file 1 [file ijms-24-13543-s001.zip › ijms-2550634-supplementary.pdf]
